# Emotional Eating and Body Misperception Among Dental Students From Romania–Implications for Targeted Interventions

**DOI:** 10.1002/hsr2.70451

**Published:** 2025-03-02

**Authors:** Denis Mihai Serban, Ancuta Mioara Banu, Costela Lacrimioara Serban

**Affiliations:** ^1^ Department of Obstetrics‐Gynecology, Discipline of Obstetrics‐Gynecology II “Victor Babes” University of Medicine and Pharmacy Timisoara Romania; ^2^ Department 2, Discipline of Maxillo‐Facial Surgery, Faculty of Dental Medicine “Victor Babes” University of Medicine and Pharmacy Timisoara Romania; ^3^ Department of Functional Sciences, Discipline of Public Health, Center for Translational Research and Systems Medicine “Victor Babes” University of Medicine and Pharmacy Timisoara Romania

**Keywords:** body perception, dental students, emotional eating, excess weight, weight status

## Abstract

**Background and Aims:**

Emotional eating and body weight misperception are significant public health issues, particularly among young adults. This study aimed to evaluate the prevalence of emotional eating and body misperception among dental students and explore the associations between emotional eating, body misperception, and sociodemographic factors.

**Methods:**

Utilizing the Emotional Eating Questionnaire and Pictorial Body Image Instrument, the study involved 300 dental students, of which 21.6% were male, with an obesity prevalence of 9.7%.

**Results:**

We found a high prevalence of emotional eating, with 58.3% of students falling into emotional and very emotional eating categories. Univariate analysis revealed that female students exhibited higher emotional eating scores and stress levels compared to males. Additionally, 34.3% of students misclassified their body weight, with 24.0% overestimating and 10.3% underestimating their weight. The multivariate model identified significant associations between high emotional eating scores and female gender (OR = 5.488, 95% CI: 2.340–12.873), body perception discrepancies, and BMI (OR = 1.194, 95% CI: 1.115–1.279), while controlling for age, perceived health status, and stress. Perceiving oneself as heavier than actual weight significantly increased the odds of high emotional eating (OR = 2.976, 95% CI: 1.623–5.456).

**Conclusion:**

This study underscores the importance of addressing emotional eating and body weight misperception among dental students to promote healthier eating behaviors, achieve leaner body mass, improve mental health, and enhance overall well‐being.

## 1 Introduction

Emotional eating (EE), characterized by the consumption of food in response to emotional cues rather than physical hunger, is a behavior that has caught significant attention in the field of public health and psychology. Individuals who engage in emotional eating are more likely to consume hyperpalatable, energy‐dense foods, leading to a positive energy balance and subsequent weight gain. From a nutritional standpoint, emotional eating disrupts the balance of a healthy diet, leading to an inadequate intake of essential nutrients and fostering a positive energy balance through the overconsumption of unhealthy foods [1, 2, 3].

Emotional eating is a common behavior that involves using food as a way to cope with negative emotions or stress. This coping mechanism is often deeply influenced by cultural and social contexts, where the relationship between food and emotions is intertwined through socialization processes. People may turn to food as a source of comfort or distraction in response to various emotional challenges, and this behavior can have a significant impact on overall well‐being and mental health [1, 4, 5].

Prior research has indicated that emotional eating is more prevalent among certain demographic groups such as young adults and females [6] and is often linked to higher BMI [2], depression [2, 7], inadequate sleep [8] and poor body image [9, 10].

However, there is limited evidence on how these factors interact specifically within the context of dental students, a population that faces unique academic and professional pressures. By focusing on this group, the study aims to fill a gap in the literature and provide insights that could inform targeted strategies for managing emotional eating in similar high‐stress environments.

This study aims to evaluate the levels of emotional eating and differences in body weight status perception among dental students. It also seeks to explore the association between emotional eating, body misperception, and the sociodemographic factors within this population. Understanding the factors associated with emotional eating is crucial for developing effective interventions, promoting healthier eating behaviors, and enhancing emotional well‐being, ultimately improving health and quality of life.

## 2 Materials and Methods

### 2.1 Participants

Using an online calculator, we determined that a minimum of 263 respondents would be needed to achieve a 95% confidence level with a 5% margin of error from the total enrollment of about 830 students in the Romanian section of the Faculty of Dental Medicine at Victor Babes University of Medicine and Pharmacy in Timisoara, Romania.

### 2.2 Procedure

The investigation first received approval from the Ethics Committee of Victor Babes University of Medicine and Pharmacy Timisoara, Romania, under number 21/29.03.2024. We conducted a stratified sampling with 50 students from each of the 6 academic years during the second semester of the 2023–2024 academic year, resulting in a sample of 300 students. The link to the questionnaire was made available on a university‐provided learning platform. The study's objectives and anticipated outcomes were detailed in the announcement, and participation was incentivized by offering a score for the Emotional Eater upon form submission. Only the first 50 students from each academic year who completed the survey were included in the analysis. The data used for this manuscript is part of a larger project aimed at understanding and improving the well‐being of dental students.

For data collection, we used an online instrument based on Google Forms. The instrument included an informed consent statement on the initial page. Participants had to acknowledge their consent before proceeding and were required to complete all questions to submit the form, ensuring no incomplete responses. Consequently, the final database was entirely free of missing data.

### 2.3 Instruments

The investigation included The Emotional Eater Questionnaire [11] previously validated in Romanian by Putnoky et al. [6] and sociodemographic questions.

### 2.4 Measurements and Data Management

The Emotional Eating Questionnaire (EEQ) is a validated tool designed to assess the extent to which individuals use food as a coping mechanism for emotional states rather than physical hunger. It captures various dimensions of emotional eating, such as disinhibition, type of food and guilt by evaluating responses to emotional triggers, such as stress, sadness, or frustration, that lead to increased food consumption. For quantifying the score of the EEQ, we followed the original scoring instructions provided by Garaulet [11], each of the 10 items was rated on a scale from 0 to 3, where 0 = Never, 1 = Sometimes, 2 = Generally, and 3 = Always. Possible scores ranged from 0 to 30 and higher total scores indicate a greater degree of emotional eating.

The survey employed diverse questions to collect information from participants, covering their gender, year in dental school, age, weight, height, and perceived stress levels. Stress was rated on a scale from 1 to 10, with higher scores indicating greater stress. Additionally, students were asked to assess their health status using a five‐point scale ranging from excellent to poor.

Students assessed their perceived body image using the Pictorial Body Image Instrument [12], a well‐established tool for measuring body image dissatisfaction. This instrument includes a series of nine drawings for both males and females, depicting adults of varying sizes from very thin (numbered 1) to obese (numbered 9). Images 1 and 2 were classified as underweight, images 3 and 4 as normal weight, images 5–7 as overweight, and images 8 and 9 as obese [12].

Body mass index (BMI) was calculated by dividing weight (in kilograms) by height (in meters) squared. BMI was categorized into weight status groups: underweight, normal weight, overweight, or obese.

Differences in body perception were determined by calculating the difference between BMI categories and perceived body categories. A score of zero indicated satisfaction with their body size, a positive score indicated that they perceived thinner, and a negative score indicated that students perceived themselves with higher weight than indicated by BMI status.

Health status was categorized as either excellent or very good perceived health versus all other levels. EE scores were divided into tertiles, with the highest tertile indicating high levels of EE.

Categorical data are presented as percentages and counts. Continuous data were assessed for normality using the Kolmogorov‐Smirnov test and were found to be non‐normally distributed. Therefore, the median and interquartile range (IQR) were used to describe central tendency and spread. The Mann‐Whitney test was employed to compare continuous variables across dichotomous factors. Logistic regression was utilized to predict the highest tertile of EE. All analyses were conducted using IBM SPSS 21. Hypothesis tests were two‐sided, with statistical significance set at *p* < 0.05.

## 3 Results

The study included 300 participants, with 21.7% (65) being male. No significant differences were observed in the proportions of males and females based on the year of study in dental medicine (*p* = 0.967) or age (*p* = 0.804) (Table 1).

**Table 1:** Socio‐demographic characteristics of the sample (*N* = 300) per gender and total.

Characteristic		Male (*N* = 65)	Female (*N* = 235)	*p* value	Total (*N* = 300)
Years of study in dental medicine	1	11 (16.9%)	39 (16.6%)	0.967	50 (16.7%)
	2	7 (10.8%)	43 (18.3%)		50 (16.7%)
	3	14 (21.5%)	36 (15.3%)		50 (16.7%)
	4	14 (21.5%)	36 (15.3%)		50 (16.7%)
	5	9 (13.8%)	41 (17.4%)		50 (16.7%)
	6	10 (15.4%)	40 (17.0%)		50 (16.7%)
Excellent or very good perceived health status	Yes	40 (61.5%)	99 (42.1%)	0.006	139 (46.3%)
	No	25 (38.5%)	136 (57.9%)		161 (53.7%)
BMI categories	Underweight	1 (1.5%)	38 (16.2%)	< 0.001	39 (13.0%)
	Normal weight	29 (44.6%)	156 (66.4%)		185 (61.7%)
	Overweight	24 (36.9%)	23 (9.8%)		47 (15.7%)
	Obese	11 (16.9%)	18 (7.7%)		29 (9.7%)
Perception of BMI categories	Underweight	3 (4.6%)	30 (12.8%)	0.001	33 (11.0%)
	Normal weight	26 (40.0%)	124 (52.8%)		150 (50.0%)
	Overweight	26 (40.0%)	62 (26.4%)		88 (29.3%)
	Obese	10 (15.4%)	19 (8.1%)		29 (9.7%)
Emotional eater status	Nonemotional eater	13 (20.0%)	27 (11.5%)	0.006	40 (13.3%)
	Low emotional eater	22 (33.8%)	63 (26.8%)		85 (28.3%)
	Emotional eater	28 (43.1%)	116 (49.4%)		144 (48.0%)
	Very emotional eater	2 (3.1%)	29 (12.3%)		31 (10.3%)
Differences in body perception	Higher than real	13 (20.0%)	59 (25.1%)	0.013	72 (24.0%)
	Accurate	37 (56.9%)	160 (68.1%)		197 (65.7%)
	Lower than real	15 (23.1%)	16 (6.8%)		31 (10.3%)
Age (years)		22.0 ± 2.0	22.0 ± 3.0	0.804	22.0 ± 3.0
BMI (kg/m²)		25.2 ± 4.6	21.1 ± 4.7	< 0.001	22.0 ± 5.5
Perceived stress levels (10 levels)		5.0 ± 3.0	7.0 ± 3.0	0.001	7.0 ± 3.5
EE score		10.0 ± 7.0	13.0 ± 9.0	0.001	13.0 ± 9.0

Among all students, 46.3% (139) reported excellent or very good perceived health status, with a higher proportion of men reporting excellent or very good perceived health status compared to women (*p* = 0.006). Regarding body weight, 25.3% (76) of the participants were classified as having excess weight, while 39.0% (117) perceived that they had excess weight. Notably, 65.7% of students had an accurate perception of their body weight, whereas 24.0% (72) perceived themselves as having higher weight and 10.3% (31) perceived themselves as having lower weight than their actual weight. Higher proportions of women perceived themselves as higher than real, as compared to men (*p* = 0.013) (Table 1).

The median BMI was 22.0, with men exhibiting higher BMI compared to women (*p* < 0.001).

Of all students, 31 (10.3%) are in the very emotional eater category while 40 (13.3%) are in the nonemotional eater category. The median (IQR) for the emotional eater score for the sample was 13.0 ± 9.0. Women had significantly higher scores in EE (13.0 ± 9.0), compared to men (10.0 ± 7.0) *p* = 0.001 and are more likely to be in the high categories of emotional eater (*p* = 0.006) (Table 1).

Data in the text represents numbers and percentages *n* (%) for categorical variables or medians +/− interquartile range for numerical variables. *p* values were obtained with Mann‐Whitney test, *p* < 0.05 were considered statistically significant

Using the BMI categories and the perceived weight status, an accuracy of classification of 65.67% was computed (197 of 300) with kappa = 0.448 and *p* < 0.001.

Table 2 presents the univariate analysis, using the High EE score (no vs. yes) as a factor. A higher proportion of females had high EE scores compared to males (87.9% vs. 12.1%, *p* = 0.005).

**Table 2:** Characteristics of participants with high EE scores (*N* = 300).

Characteristic		No High EE Score (1–15) (*N* = 201)	High EE Score (16–28) (*N* = 99)	*p* value
Gender	Male	53 (26.4%)	12 (12.1%)	0.005
	Female	148 (73.6%)	87 (87.9%)	
Excellent or very good perceived health status	Yes	104 (51.7%)	35 (35.4%)	0.008
	No	97 (48.3%)	64 (64.6%)	
Differences in body perception	Higher than real	38 (18.9%)	34 (34.3%)	0.029
	Accurate	143 (71.1%)	54 (54.5%)	
	Lower than real	20 (10.0%)	11 (11.1%)	
Age (years)		22.0 ± 3.0	22.0 ± 3.0	0.295
BMI (kg/m²)		21.3 ± 5.1	23.2 ± 6.6	< 0.001
Perceived stress levels (10 levels)		7.0 ± 4.0	7.0 ± 3.0	0.067
EE score		9.0 ± 6.0	19.0 ± 4.0	< 0.001

Participants with high EE scores reported lower levels of excellent or very good perceived health status (35.4% vs. 51.7%, *p* = 0.008) and higher BMI (23.2 vs. 21.3, *p* < 0.001). Those with high EE scores were more likely to have a higher perceived body weight than their actual body weight (34.3% vs. 18.9%, *p* = 0.029). There was no significant difference in age (*p* = 0.295) and perceived stress levels (*p* = 0.067) between participants with and without high EE scores.

Data in the text represents numbers and percentages *n* (%) for categorical variables or medians +/− interquartile range for numerical variables. p‐values were obtained with the Mann‐Whitney test, *p* < 0.05 were considered statistically significant

A logistic regression analysis was performed to determine the predictors of high EE scores among the participants. The results are presented in Table 3. Female participants had significantly higher odds of having high EE scores compared to male participants (OR = 5.488, *p* < 0.001), indicating that females were more likely to exhibit high emotional eating behavior. Participants who perceived their body weight as higher than actual had significantly higher odds of having high EE scores compared to those with accurate perception (OR = 2.976, *p* < 0.001). However, perceiving body weight as lower than actual was not a significant predictor (OR = 1.400, *p* = 0.499).

**Table 3:** Logistic regression analysis for predictors of high emotional eating scores.

Predictor	OR	95% CI for OR	*p* value
Female gender versus male gender	5.488	2.340–12.873	< 0.001
Excellent or very good perceived health status (yes vs no)	0.763	0.429–1.356	0.357
Age (years)	1.021	0.906–1.151	0.729
Perceived stress levels	1.031	0.907–1.173	0.640
Differences in body perception			0.002
Lower than real versus accurate	1.400	0.528–3.710	0.499
Higher than real versus accurate	2.976	1.623–5.456	< 0.001
BMI (kg/m²)	1.194	1.115–1.279	< 0.001

Higher BMI was a significant predictor of high EE scores (OR = 1.194, *p* < 0.001), indicating that participants with higher BMI were more likely to have high emotional eating behavior.

Perceived health status, age, and perceived stress levels were not significant predictor of high EE scores but were kept in the model, controlling for their effects.

Dependent variable: highest tertile of EEQ score.

Independent variables: Female gender (vs. male gender), Excellent or very good perceived health status (vs. lower levels), Perceived stress level (10 levels), Differences in body perception (3 levels, Lower than real vs. accurate Higher than real vs accurate).

## 4 Discussion

This study evaluated emotional eating levels and body weight perception among dental students, exploring their associations with sociodemographic factors. To our knowledge, this is the first study to investigate these relationships in a dental student population.

A notable 58.3% of participants exhibited emotional eating behaviors, consistent with findings by Frayn et al. [13], who reported that emotional eating is predominantly triggered by internal cues, such as stress and anxiety, rather than external stimuli. Women showed significantly higher emotional eating scores and stress levels compared to men, reflecting societal pressures disproportionately affecting female students [5, 14, 15]. High academic demands likely amplify these behaviors, as suggested by previous studies linking stress to maladaptive eating in student populations [2, 14].

Emotional eating has been associated with binge eating disorder and subsequent weight gain [1, 16]. For example, Kells [17] reported a 31% prevalence of binge eating disorder in female college students. In our study, 12.3% of women and 3.1% of men fell into the “very emotional eating” category, putting them at heightened risk for binge eating and weight gain [18]. Multivariate analysis revealed significant predictors of high emotional eating, including female gender (OR = 5.488, 95% CI: 2.340–12.873) and BMI (OR = 1.194, 95% CI: 1.115–1.279), aligning with studies by Madalı et al. [19] and Vasileiou and Abbott [20], which demonstrated stronger associations between emotional eating and obesity.

A recent study on Romanian medical students [21] revealed that excess weight significantly predicted lower levels of mindful eating—defined as eating with awareness and without judgment—while the emotional response was higher in students who gained over 5 kg in the past year. While examining emotion regulation as a moderator, Barnhart [22] demonstrated that higher levels of negative EE correlated with weight concerns and elevated disordered eating scores when emotion regulation difficulties were average or higher. Another study [23] found a significant association between body image dissatisfaction and symptoms of eating disorders, emphasizing the need for dental professionals to be aware of these issues when treating patients. Hodgson [24] highlights the effectiveness of dental undergraduate education in raising awareness about eating disorders. Incorporating comprehensive training on recognizing and managing eating disorders can better prepare dental students for clinical practice and self‐awareness.

Our research revealed a notable disparity between perceived and actual body weight status, with over a third of students (34.3%) inaccurately assessing their weight. The 0.448 coefficient of agreement (kappa) between perceived body category and BMI‐derived status suggests a moderate level of agreement [25]. Among the students, 24.0% (72 students, of whom 59 were women) overestimated their body size, while 10.3% (31 students, of whom 16 were women) underestimated it. Misjudgment of body weight may either lead to neglect of genuine health concerns or unwarranted distress over perceived overweight [10, 26, 27]. Robinson [26] noted that the normalization of larger body sizes contributes to the underdetection of obesity, delaying interventions. Additionally, Stunkard et al. [28] found that distorted body perceptions were more prevalent in individuals who were obese during childhood, negatively impacting their interpersonal relationships and self‐image. On the other hand, the pressure to conform to unrealistic body standards driven by peer influence can lead to distorted body image perceptions and an increased risk of developing body image issues and eating disorders. This is especially true during adolescence and young adulthood, when peer acceptance plays a crucial role [29, 30, 31].

A significant association was observed between body weight overestimation and high EE (OR = 2.976, 95% CI: 1.623–5.456). This aligns with Shriver et al. [32], who reported that body dissatisfaction exacerbates emotional eating, particularly in women. Interventions promoting intuitive eating, which foster body appreciation and positive psychological constructs, have been effective in reducing emotional eating [33]. Moreover, Linardon et al. [33] highlighted that emotion regulation strategies mitigate disordered eating behaviors, particularly when individuals struggle with stress and body image concerns.

In the dental student population, perceiving body weight as thinner than it actually is was not statistically significantly associated with EE (OR = 1.400, 95% CI: 0.528–3.710). This finding may be explained by the fact that this type of body misperception is more common in men [34], who are generally less affected by emotional eating [2].

Dental students face considerable stress from academic workload, examination anxiety, grading concerns, and clinical responsibilities [35, 36]. These stressors impact academic performance and emotional well‐being, with cultural and ethnic factors further modulating stress responses across student populations [36]. Stress is also linked to maladaptive coping behaviors such as smoking, substance use, and, in non‐health‐related disciplines, emotional eating [35, 37]. However, consistent with our findings, this association was not observed among students in health‐related fields [37].

Contrary to some prior findings, no significant association was observed between perceived stress and emotional eating in our population. Devonport et al. [38] similarly noted that stress influenced eating behaviors during exam periods but not in routine contexts. Queirolo et al. [39] reported that dental students experience moderate levels of stress, anxiety, and burnout, which significantly impact their executive functions. Adequate rest was found to buffer these effects, highlighting the importance of self‐care in this population.

Research from 2024 [40] evaluated dental students' lifestyle behaviors, physical activity levels, and social media use. Findings suggest that high social media usage correlates with unhealthy lifestyle choices, potentially exacerbating stress and emotional eating behaviors. A long‐term study [41] highlighted the enduring health implications of disordered eating behaviors observed in individuals in their mid‐20s. Such behaviors were found to predict poor self‐rated health and psychological distress a decade later, emphasizing the critical need for early interventions.

Intervention strategies such as cognitive‐behavioral therapy (CBT) have shown promise in addressing emotional eating and improving body image [42]. Weight‐loss programs incorporating CBT effectively reduce emotional eating, particularly in individuals with higher BMI. Johnson et al. [43] demonstrated the potential of integrating artificial intelligence into CBT to enhance accessibility and effectiveness in managing stress and disordered eating among students. Similarly, culturally tailored interventions, such as adherence to the Mediterranean diet combined with awareness campaigns, have improved body image and reduced disordered eating behaviors [44]. For body image disturbances, interventions such as mirror exposure therapy and third‐wave behavioral approaches (e.g., mindfulness‐based therapy and acceptance and commitment therapy) are effective in improving body satisfaction and reducing the risk of eating disorders [45, 46].

Despite its contributions, this study has limitations. Its cross‐sectional design precludes causal inferences, and reliance on self‐reported data may introduce bias. Longitudinal research is essential to establish causality and explore additional factors influencing emotional eating, such as personality traits and social support systems.

In conclusion, this study highlights significant patterns of emotional eating and body weight misperception among dental students. Women demonstrated higher emotional eating scores and a greater tendency to overestimate their weight. Emotional eating was strongly associated with BMI and body weight misperception but was independent of perceived stress. These findings underscore the need for targeted, evidence‐based interventions to address emotional eating and body image concerns in this population.

## Author Contributions

**Denis Mihai Serban:** formal analysis, writing – original draft, validation, conceptualization. **Ancuta Mioara Banu:** software, validation, formal analysis, investigation, writing – review and editing, conceptualization. **Costela Lacrimioara Serban:** conceptualization, methodology, writing – review and editing, supervision.

## Ethics Statement

The study was conducted in accordance with the Declaration of Helsinki, and approved by Ethics Committee of Victor Babes University of Medicine and Pharmacy Timisoara, Romania, under number 21/29.03.2024.

## Consent

Informed consent was obtained from all subjects involved in the study.

## Conflicts of Interest

The authors declare no conflicts of interest.

## Transparency Statement

The lead author Ancuta Mioara Banu affirms that this manuscript is an honest, accurate, and transparent account of the study being reported; that no important aspects of the study have been omitted; and that any discrepancies from the study as planned (and, if relevant, registered) have been explained.

## Data Availability

The data that support the findings of this study are available from the corresponding author upon reasonable request.
